# Development and Operation of an Experimental System to Measure the Moments Generated in the Finger Joints

**DOI:** 10.3390/bioengineering9050184

**Published:** 2022-04-22

**Authors:** Gregory Chagnon, Khouloud Achouri, Nathanael Connesson, Julien Gerbelot, Aurelien Courvoisier

**Affiliations:** 1 Université Grenoble Alpes, CNRS, UMR 5525, VetAgro Sup, Grenoble INP, TIMC, 38000 Grenoble, France; khouloud.achouri@univ-grenoble-alpes.fr (K.A.); nathanael.connesson@univ-grenoble-alpes.fr (N.C.); 2 Demeure Orthopédie, 38400 Saint-Martin d’Hères, France; jgerbelot@demeureorthopedie.fr; 3 Université Grenoble Alpes, CNRS, UMR 5525, VetAgro Sup, Grenoble INP, CHU Grenoble Alpes, TIMC, 38000 Grenoble, France; acourvoisier@chu-grenoble.fr

**Keywords:** finger, experimental device, experimental measure, articulation, principal component analysis

## Abstract

Little information is available on the forces that fingers can generate, and few devices exist to measure the forces they can create. The objective of this paper is to propose an experimental device to measure the moments generated by finger joints. The idea is to focus on a single joint and not on the effort generated by the whole finger. A system leaving only one joint free is developed to measure the maximum attainable moment in different joint positions between the extended and flexed finger. The device is tested on the proximal interphalangeal joints of the index fingers of thirty people for both hands. The results show a dispersion of results from one person to another but with similar trends in the evolution of the maximum achievable moment depending on the angle. Average values of the maximum moments attained by men and women for both hands are given for all angular positions of the joint. The results are analysed using principal component analysis. This analysis shows that four main modes represent more than 99% of the signal and allow the reconstruction of all the data for all the subjects. The four modes obtained can be used as a basis for the development of finger devices by hospital practitioners.

## 1. Introduction

The hand is the effector organ of the upper limb and is a complex tool with a large number of degrees of freedom. It has two main functions of great precision: grasping and tacting [1]. It is composed of several series of small bones that form the carpus, metacarpus and phalanges. Each finger has three phalanges, except for the thumb, which has only two. The phalanges are named P1, P2 and P3 in the proximal to distal direction. The phalanges are connected to each other by movable joints, controlled by long tendons that form the end of the arm muscles. The joints of the hand include: the carpometacarpal joints, the metacarpalphalangeal joints and the interphalangeal joints. These joints link the phalanges of the hand together. The index, middle, little and ring fingers each have two joints: the proximal interphalangeal (PI) and distal interphalangeal joints. Only one degree of freedom (flexion/extension) is allowed for these joints, which permits the hand to be closed and a grip to be made.

The movements of the hand are the result of a balance between a set of agonistic and antagonistic and extrinsic and intrinsic muscles. The extrinsic muscles, located in the forearm, transmit movement to the hands and fingers via long tendons that run either along the palm (flexor tendons) or along the back of the hand (extensor tendons). The intrinsic muscles, located in the hand, transmit the precise movements of the fingers. The interosseous muscles are distinguished according to their location, dorsal (back of the hand) or palmar (palm), and allow the fingers to be moved apart and together respectively. The lumbar muscles, present between each of the five fingers, participate in flexion and extension, while the thenar muscles are used to move the thumb and the hypothenar muscles to move the little finger. All of these muscles and tendons generate bending forces in the fingers.

The measurements of the forces generated in the joints are important data for many applications: the understanding of pathologies; the optimisation of treatments, for example, for ageing [2]; the design of ortheses and prostheses; the reproduction of movements or the understanding of gestures; the impact on blood circulation [3]; the creation of bionic hands [4], or the development of an exoskeleton [5]. The knowledge of the moment in the joint is the first step towards the estimation of the tensions on the tendons that cross this joint. In this context, several devices have been developed to provide the necessary information in terms of force and bending moment. The first means and results of measurements are available in the synthesis article [6]. Some features can be further detailed. The flexible hand glove, for example, is considered a good model for the kinematic analysis of finger joints [7], allowing for the determination of the position as well as the bending angle of each finger joint by means of (flexible) bending sensors located at the joints. Recently, gloves have been instrumented to measure the force in hands in daily activities [8]. There are various systems for measuring finger forces, but the majority of devices are designed to measure grip force [9,10] or pinch force [11] in a single joint position. Dynamometers and hydraulic pressure gauges are the best known tools for measuring these forces. Therapists ultimately make the connection between grip force and neuromuscular power. However, these simple systems do not allow a precise link between the force developed by the joint and the angle of flexion of the same joint to be assessed [11]. Different devices were developed. One was made to judge the extent of rehabilitation, which is important information for therapists to recover the initial state of the fingers. It consists of a cylindrical-type finger force measurement system with four force sensors. The system developed can measure the pinch force of each of the patient’s fingers. Other devices were designed to measure finger forces in different wrist movements: extension/flexion or adduction/abduction of the wrist [12]. However, these devices are not able to accurately measure the force as well as the bending moment of the finger joints in each joint angle. A force measurement system called Dyna-8 [13] was developed and used to determine hand and finger forces in model situations. It is equipped with transducers to measure grip force and finger forces. These devices do not take into account the bending angle, providing no information of the bending moment and limited data regarding the force measurements. Similarly, Kilgore et al. [14] created an experimental device capable of simultaneously measuring the isometric moments generated in the metacarpophalangeal, proximal interphalangeal and distal interphalangeal joints of all four fingers. The device consists of four aluminium bars that are attached to each segment of the joints as well as the dorsal part of the hand and can be adapted to different hand sizes. The system is designed to act as a splint, allowing each joint to be positioned at different angles of flexion. However, the use of this device is limited for several reasons: it has a large mass (2.2 kg) and the forces of the straps holding the fingers disturb blood circulation, making the system uncomfortable for the user. A different idea based on a motorised hand exoskeleton was proposed [15]. It was designed to be partially open at the fingers to allow the user to be in direct contact with the grasped object in order to preserve the natural haptic sensation. When manipulating an object, the contact force of the finger is estimated from the measurement made by a tactile capacitive force sensor of type FSR located at the inner surface of the finger part of the exoskeleton. The force determined is the gripping force. The influence of finger assembly on grip strength has recently been studied [16]. Fingertip moments were also measured during gripping [17]. The WFTS (Wrist Finger Torque Sensing) device measures the torque around the flexion axis of the wrist joint as well as the metacarpophalangeal joints of the fingers [18], but there is often a problem with the alignment of the axes of the device and the fingers.

The work presented here is part of a general framework that aims to offer not only rigid or free movement orthoses to fit the patient but orthoses in which stiffness can be controlled. To achieve this objective, the first step is to be able to quantify the efforts that can be generated by each individual. The various devices created allow certain mechanical quantities to be measured, but they still have limitations. The objective here is to be able to measure maximum moments in all angular positions of the finger. Thus, the aim of this article is to propose a new device that meets this need and is also ergonomic and easy to use. A proposal for a new device is presented in Section 2, in which the method of use, calibration and technical details are outlined. The system is then tested on 30 subjects, and the results are presented in Section 3. These results are then analysed by principal component analysis and discussed in Section 4.

## 2. Materials and Methods

### 2.1. Experimental Development

**Figure 1:**
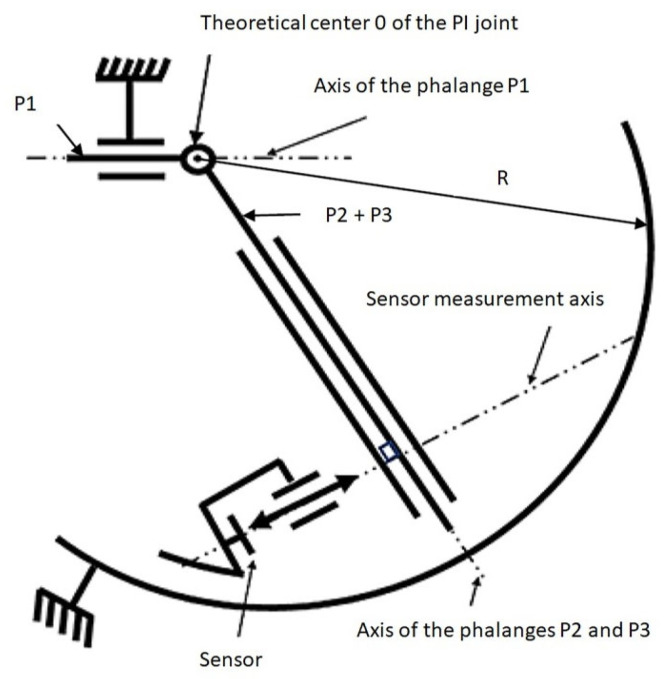
Kinematic diagram of the force measurement system for the finger joints.

The objective is to develop a device capable of measuring the maximum bending moment of the PI joint in different angular configurations. The system must be able to adapt to different finger sizes, even though these may vary significantly [19]. In this objective, the device must be able to give three quantities: the maximum force, the centre of the PI joint and, finally, the point of application of the force. A kinematic diagram of the device is proposed in Figure 1. The objective is to have the centre of rotation of the PI joint on point O. To do this, phalange P1 should be placed on the horizontal axis and phalanges P2 and P3 should be positioned in line but in a different direction. The angle is controlled at the centre of rotation. The axis of the phalanges applies its force at a known point which is in contact with the sensor to compute the maximum moment generated in the joint. The whole system is placed in an arc where the angular position can be adjusted and therefore the choice of measurement configured.

**Figure 2:**
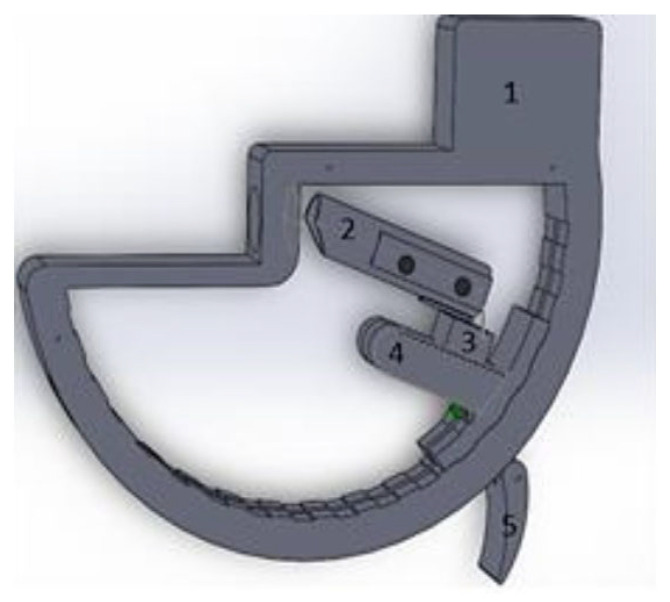
Experimental system of effort measurements. 1—system body; 2—rigid cylindrical tube; 3—sensor mounting; 4—T-piece; 5—hanging flange.

A representation of the system is given in Figure 2. The different parts of the device are made by 3D printing. The body of the system is rigid and has a shape that allows the user to place their finger inside so that the proximal phalange is fixed on the arch. The hand can rest on the system during manipulation. The medial and distal phalanges are then inserted into a rigid cylindrical tube (part 2) resting on the sensor (part 3) which can be fixed in different angular configurations by means of the T-piece (part 4) that can be fixed in any position by the hanging flaugue (part 5). The tube can have different relative positions with respect to the sensor, without affecting the measurements, due to the differing sizes of the subjects’ fingers and the differing ways the fingers are pressed into the tube. The important point is that the joint of the phalanges P2 and P3 remains well inside the cylinder to block any rotation movement. The system is dimensioned with respect to the articular limits of the PI [0°, 110°] and with respect to the geometry of the finger (diameter and length of the phalanges). Foam parts can be added to the hole and cylinder to fit the specific shape of the finger.

**Figure 3:**
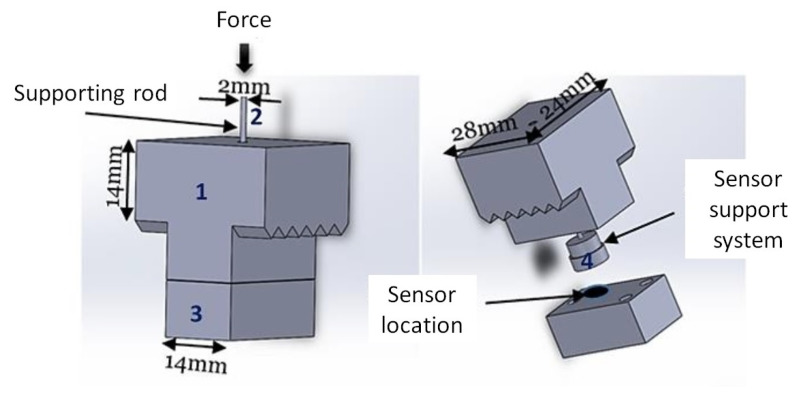
System to support the sensor.

Various means of measurement are used to instrument the system. Two black targets are added to the tube to follow the movement of the finger for the determination of the centre of the PI joint. The tube rests on the sensor assembly which is further described in Figure 3. The sensor assembly (part 3 of Figure 2) is detailed in Figure 3 and consists of four small components, the first of which being a top part into which a 2 mm diameter support rod is inserted, which is in direct contact with the cylindrical tube via the groove in the tube. The end of the inserted rod then rests on a small cylindrical body (support system) located inside the sensor assembly, which is in direct contact with the sensor. It should be noted that the active surface of the sensor is covered by foam in order to have a uniform distribution of the applied force. In this configuration, the force is always perpendicular to the sensor. The sensor is a miniaturised Singletact capacitive force sensor. The sensor was chosen for its ergonomic advantages due to its small size of 58 mm in length and 8 mm in diameter but also for its measuring range of approximately 30 N. The FSR sensor measures only the force component in the rod direction assuming neglectable friction.

### 2.2. Calibration of the Device

The FSR sensor is placed in the sensor system together with the support system so that the measurement is taken by the circular part, visible in Figure 3, resting on the FSR sensor. The circular part is set in motion by the supporting rod which receives the force. It was decided to calibrate the sensor in the configuration in which it would be used. Thus, the whole system was calibrated by known masses between 100 g and 3 kg placed on the sensor system. The repeatability of the measurements was also validated during the calibration of the sensor.

**Figure 4:**
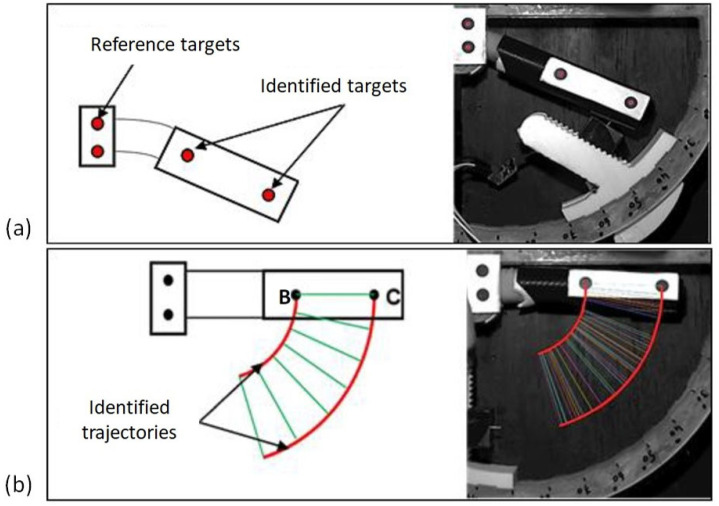
Optical instrumentation of the device with the use of circular targets. (**a**) Configuration of the device for measuring forces, (**b**) configuration without sensor for determining the centre of rotation of the joint.

Before performing the stress tests, a pretest is necessary to locate the centre of rotation of the PI joint. This test should be systematised to reduce error since the position of the centre of rotation depends on the morphology of each finger and the positioning of the finger in the device. Two circular targets are set at the tube (part 2). Two other targets are also placed on the rigid body to define a reference for the system as illustrated in Figure 4. In this step, the subject comes to place his/her finger in the system without the sensor system mounted as shown in Figure 4b. This allows the finger joint to rotate without obstruction. A flexion-extension movement at the PI joint is generated by the subject. During this back-and-forth movement of the finger, pictures are taken automatically every 100 ms using a CDD camera with a sensor of 1028 × 1028 pixels. This allows the position of the two markers placed on the device and thus their trajectories to be acquired at any time. The rotational movement of the joint theoretically generates circular translational movements of the markers. The data are processed with MATLAB and the trajectory circles are identified in the least-squares sense. The knowledge of the circles finally allows for the determination of the centres of rotation of the two identified markers. An illustration of the arcs of the circles is presented in Figure 4b. The joint centre is then determined as the average of the two identified centres. It should be noted that the maximum deviation between two identified centres must be less than or equal to 2 mm, which represents approximately 4% of the length of the lever arm; otherwise, the test must be repeated to minimise errors. The deviations are due to the fact that no finger guidance is conducted in the plane, so a slight out-of-plane movement could take place. A certain amount of freedom of movement of the finger is also possible and induces some errors. The nonsystematic use of cameras leads to the assumption that the centre of rotation is known in its theoretical position, which can lead to significant uncertainty. It is therefore preferable to use them for each subject.

**Figure 5:**
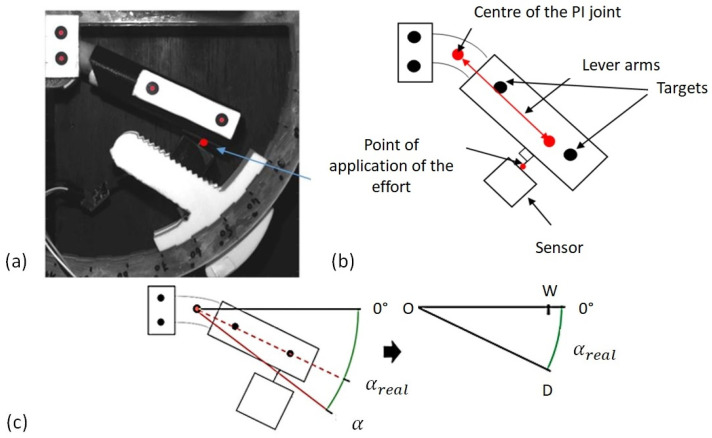
Setting up of the system and means of measurement. (**a**) Positioning of the targets and the point of application of the force, (**b**) determination of the lever arm between the centre of rotation and the point of application of the force, (**c**) evaluation of the real angle during the test.

Once the centre of rotation is identified and the T-piece is fixed at an angular position α, the maximum force tests can be performed. In practice, the true angle is not exactly that of the device as illustrated in Figure 5, because the centre of rotation of the finger is not the same as the theoretical centre of the device. The real angle can be obtained simply as a function of the coordinates of points D and O in the system reference frame:

(1) $$\tan\alpha_{real}=\frac{y_{D}-y_{O}}{x_{D}-x_{O}}$$

where $x_{O}$, $y_{O}$, $x_{D}$ and $y_{D}$ are the Cartesian coordinates of the points *O* and *D*. This information is simply obtained by capturing an image at the beginning of each stress test. The angular positions are thus corrected for each measurement to have a correctly evaluated couple (position, moment).

The sensor measures the contact force at the point shown in Figure 5b. The orientation of the force is given by the axis of the rod on which the finger applies the force. Since the centre of rotation has been identified in the calibration stage, it is possible to determine the lever arm between the point of force application and the centre of rotation. Finally, the moment in the joint is simply obtained by the vector product of the force and the lever arm vector.

### 2.3. Test Plan

**Figure 6:**
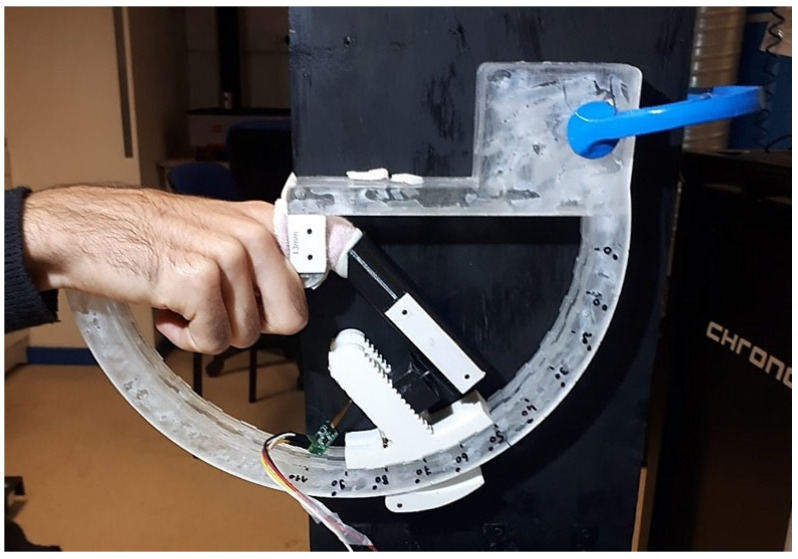
Hand positioned in the experimental device for a test with an angle of α=50.

A series of tests were carried out on the index finger of both hands of 30 healthy human subjects. The group was composed of 18 men and 12 women, and the average age was 31, the youngest being 20 and the oldest 62. The profiles of the subjects were very different between sportsmen/sportswomen and nonsportsmen/nonsportswomen. The subjects had very different body mass indexes but were all in good health, and none of them had any pathology in their hands or fingers. The right hand for right-handed people and the left hand for left-handed people was called the hand H1, the other hand was called hand H2. In order to avoid the intervention of other muscles such as the biceps or triceps during the application of force, the subject was placed in a seated position with his/her arm supported for each test. The subject inserted his/her hand into the device. A first empty movement was performed to locate the centre of rotation of the joint. Although the PI joint can reach 100 or even 110 degrees for some subjects, beyond 90 degrees it becomes difficult to generate a significant force and the position in the system can become uncomfortable. It was therefore decided to limit the test to the 0–90 range. Measurements were taken every 10 degrees. The tests were performed on both hands for each subject. An illustration of a hand in the experimental device is presented in Figure 6.

### 2.4. PCA Analysis

Biomechanical data are often complex, and a good tool to process these data is principal component analysis (PCA), which is used in particular for the study of joint movements [20]. Limitations of the technique have been highlighted [21]; however, it remains an effective tool and is applied here to the moment measurements made. The data are highly dependent on the physical strength of each individual. As the objective here is to evaluate the evolution of strength according to the angle of the joint, the data are centred and reduced by looking at the evolutions around the average. This aims to give the measurements the same weight in the analysis to each subject. Let *m* be a measurement performed, $m_{moy}$ its mean and σ its standard deviation for all subjects. The centred reduced value is then defined by:

(2) $$m^{*}=\frac{m-m_{moy}}{\sigma }$$

The analysis is carried out for all the measured moments and for each of the two hands independently so that two analyses are proposed on H1 and H2. The PCA requires an assessment of the correlation between the variables; therefore, this is assessed for all pairs of measures. Consider two measured values $m_{1}$ and $m_{2}$ for two different angles, let *i* denote the index of the *i*th measurement of these variables for a total of *p* measurements, and let $m_{1}^{i}$ and $m_{2}^{i}$ denote the measures. Thus, the correlation is defined by:

(3) $$cov\left(m_{1}^{i},m_{2}^{i}\right)=\left(\frac{1}{p}\sum_{i=1}^{p}m_{1}^{i}m_{2}^{i}\right)-m_{1moy}^{i}m_{2moy}^{i}.$$

The eigenvalues $\lambda_{k}$ and eigenvectors $F_{k}$ of the covariance matrix are obtained classically, with k=1,…,9 being the number of measurements made during the campaign. The eigenvalues are ranked from the most important to the least important, highlighting here their weight in the biomechanical response. Any measure *F* can thus be reconstructed in the main database after determining the components $\alpha_{k}$ in this database:

(4) $$F=\sum_{k=1}^{9}\alpha_{k}F_{k}.$$

## 3. Results

### 3.1. Repeatability

**Figure 7:**
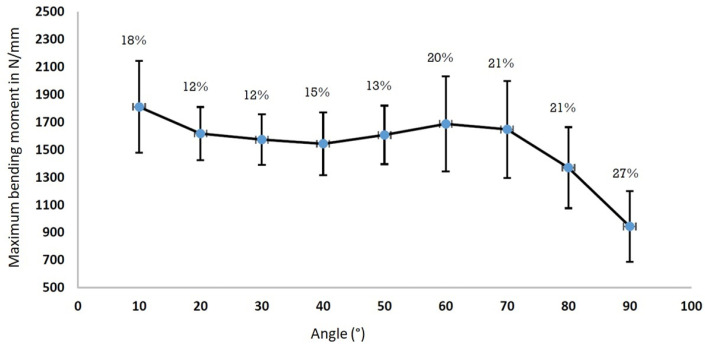
Repeatability of results on a subject. Mean value and range of variation for each measurement angle.

The same test was performed ten times for the same subject to analyse the reproducibility of the results, with these tests being performed on different days using their H1 hand. An illustration is given in Figure 7. Variations of the order of 15–20% can be noted between the values, which can be explained for multiple reasons: the experimental dispersion of the device, but also the physical condition of the subject from one day to another, the handling of the experimental device and the habits taken for the measurements. From a practical point of view, the order of magnitude of these variations is not prohibitive for measurements on a patient and allows for an exploitation of the following since the same trends were observed for all 10 tests.

### 3.2. Force Tests

**Figure 8:**
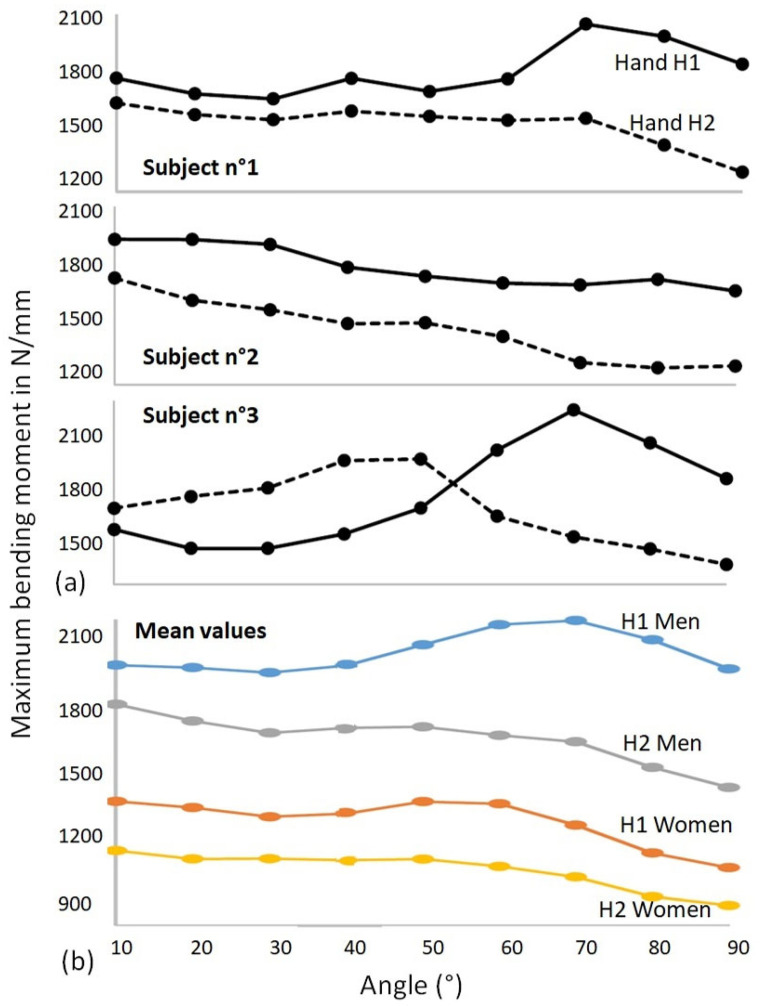
Evolution of the maximum bending moment as a function of the angular position for both hands H1 and H2 (**a**) for subjects n°1, 2 and 3; (**b**) mean values of the maximum bending moment for women and men.

Different results were observed for the 30 subjects tested; however, similar patterns frequently occurred. These are illustrated in Figure 8a with the presentation of the response curves of both hands for three different subjects.

The values obtained for the 30 subjects were averaged into four different groups, women’s and men’s measurements and H1 and H2 hand measurements. All response curves are presented in Figure 8b. It should be noted that the curves by subject show greater variations than the average curves, which smooth out these phenomena over all the subjects. For women, the average value of the moments is about 1300 N/mm for hand H1 and 1100 N/mm for hand H2, while for men the moments are about 2100 N/mm for hand H1 and 1700 N/mm for hand H2. However, these numbers change significantly depending on the angle of flexion of the finger. Variations of 20 to 30% can be observed depending on the angle of the joint.

### 3.3. PCA Analysis

The experimental data are processed for all measurements on the 30 subjects for both hands H1 and H2. The results of the correlated matrix are presented in Table 1 and Table 2 for the two hands, respectively.

These tables provide a link between all the variables and effort measurements that have been carried out and summarise all the tests conducted. These correlation matrices are used to determine the associated eigenvalues and eigenvectors in the PCA analysis. The results are given in Table 3 and Table 4 for the hands H1 and H2, respectively.

**Figure 9:**
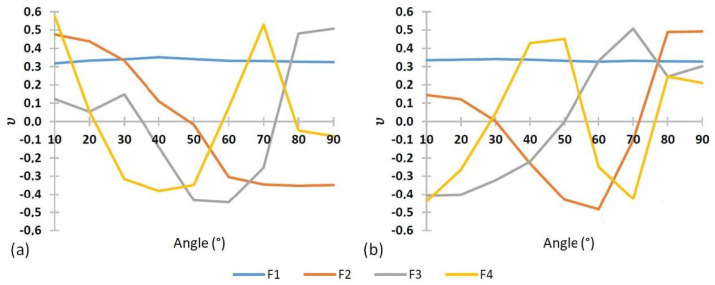
Representation of the first 4 eigenmodes for the two hands (**a**) H1 and (**b**) H2.

Firstly, it appears that the results are different for the two hands. Nevertheless, the results obtained for both hands show similar trends. The eigenvalues and their associated weights for both hands show a clear predominance of mode 1, which represents 85 to 92% of the response. For both hands, we see that four modes represent more than 99% of the response. The last five modes become almost negligible in the response. The first four modes are shown in Figure 9. Mode 1 has a horizontal line corresponding to a quasiconstant moment at any angle. The shape of the mode is quite similar to the average curves that can be seen in Figure 8b. The following three modes represent variations from this average force state and define the variations of forces according to the angles. Each of the modes has its minima and maxima at different angles. It can be observed that mode 2 of the H2 hand is very close to mode 3 of the H1 hand. The order of the mode is directly linked to the eigenvalues, explaining the different orders by their different weights. Thus, depending on the hand, the variations with respect to the state of maximum force do not have the same relative weight from one hand to the other.

## 4. Discussion

Looking at the overall results, there are several clear remarks. The results obtained depend on the subject, since the responses can be very different from one subject to another. There are also strong disparities between the two hands of the subjects. Another remark is that the maximum possible efforts to be generated depend very strongly on the angle. A maximum force is often observed for an angle value between 50 and 70° depending on the subject for one or both hands. These subjects felt more comfortable applying effort in the device and this was independent of whether the subject was a sportsman/sportswoman or not. For other subjects, a decrease in strength is observed with the angle. These observations are identical for both sexes. The values of the maximum moments depend on the strength of each individual, but the general shapes of the curves remain similar. The H1 hand is in the vast majority of cases stronger than the H2 hand, but there are exceptions, as in the case of subject n°3. The right hand for right-handed people, and the left hand for left-handed people, is not necessarily the dominant hand, since the H2 hand also has an important gripping role that can generate a lot of force. The balance is therefore different from one individual to another depending on their activities. The average curves show smaller amplitudes of variation than the curves for each subject. For the H2 hand, both men and women show a tendency for the force to decrease with the angle of flexion of the joint, whereas for the H1 hand, although there is a decrease with the angle, there is also a peak in force at around 60 or 70 degrees.

The measurements obtained with the device allow for the determination of the moments in each joint. These results differ from those that only offer measurements of global forces in the fingers [11,13,16]. The resulting global force depends on the length of the phalanges, but additional information can be obtained on the geometry of the fingers using [19]. The global measurements of pinch or grip allow us to obtain a global effort in the tendons but do not give the values in each joint; only the global efforts at the fingertips are obtained. The measurement system proposed by [18] allows for the determination of the moments in the whole of the fingers but not in a fixed angular position. Compared to another existing system, our system requires precision on the centre of rotation [14], but it has the advantage of being less cumbersome and easier to set up. Moment values obtained by [17] during a gripping effort are four times lower than those obtained in this study, but the authors were not looking for obtaining the maximum value but rather the moment during a gripping effort.

The PCA made it possible to determine four eigenmodes capable of representing 99% of the signal. Each experimental curve can thus be reconstructed by these deformation modes. The weights of each of the modes are identified for all the experimental load cases, of which the values of the parameters for hand H1 are presented in Table 5 and for hand H2 in Table 6. The weights of the modes vary from one individual to another.

**Figure 10:**
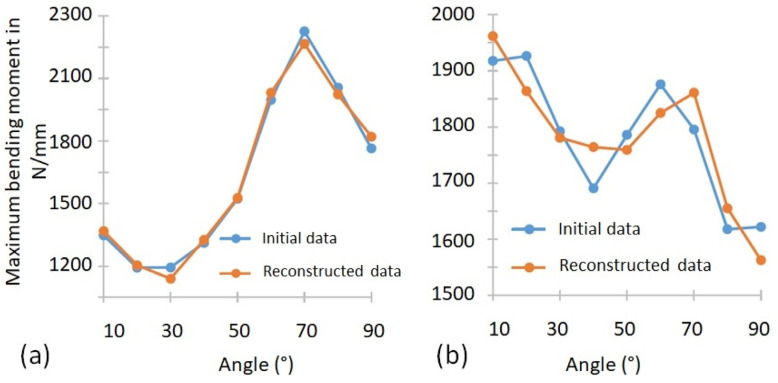
Reconstruction of the initial measurement from the 4 main eigenmodes identified for (**a**) the hand H1 and (**b**) the hand H2.

The weight of mode 1 is, for the majority of individuals, the most important weight of the reconstruction. As this mode is relatively constant with angle, it allows us to obtain an average force state for all bending angles. The subjects for whom this weight is lower correspond to the subjects where a significant decrease in moment with bending angle is observed. The relative weight of the other modes is more difficult to analyse, since these modes present strong variations according to the angle, constituting the adjustment variables which reconstruct the starting signal. The advantage of using modes is the possibility of creating a noise filter to present more regular signals. An illustration of the reconstruction of two signals is shown in Figure 10. Figure 10a shows a representative reconstruction of more than 90% of the signals where all four modes are able to reproduce the original signal almost perfectly. Figure 10b shows an example of a H2 hand where the four-mode base has the most difficulty in reconstructing the original signal. It can be seen that the signal is not perfectly described, with some variations of the moment as a function of the angle being smoothed or shifted.

Despite these deviations, it is clear that this generic basis allows for the reconstruction of new subjects and thus is an easy numerical tool for modelling moments as functions of the finger joint angle. This eigenmode base provides a tool for physicians to represent effort as a function of angle. Numerical tools can then be developed from each mode, and the result can be applied to each patient by recombining each mode contribution. In the end, this should enable the possibility of the design of orthoses which can be deformed under controlled effort through the development of models that define characteristics associated with each mode. The primary interest is to define the characteristics of the medical devices according to the properties of each mode. The measurement of a patient’s moments will have to be decomposed on the basis obtained to combine the characteristics of the medical devices for each mode.

## 5. Conclusions

This study has led to the development of a measuring device that can quantify the maximum moment in each finger joint. On a different scale, the system can be applied to any joint with a rotational degree of freedom. An application of the device was carried out on the index finger. The analyses showed different results from one subject to another, but after a principal component analysis, four eigenmodes sufficient to represent the maximum moments were identified. These allow 99% of the data to be represented and are therefore a reliable basis for the description of the moments that can be generated as functions of the angle in the PI joint.

This study shows the high interindividual and intraindividual (right versus left hand) variability of the maximum moment in the finger PI joint. From a clinical point of view, this is a plea for a patient-specific evaluation of the forces and moments in the joints in order to better understand the patient condition and conduct the appropriate treatment. This work can be used as a basis for the design of suitable medical devices.

## Figures and Tables

**Table 1:** Correlation matrix of H1 Hand.

Angle	10°	20°	30°	40°	50°	60°	70°	80°	90°
10°	1	0.958	0.916	0.856	0.780	0.696	0.728	0.705	0.697
20°	0.958	1	0.975	0.924	0.854	0.753	0.746	0.738	0.741
30°	0.916	0.975	1	0.954	0.878	0.772	0.749	0.804	0.805
40°	0.856	0.924	0.954	1	0.965	0.891	0.852	0.834	0.828
50°	0.780	0.854	0.878	0.965	1	0.940	0.886	0.786	0.775
60°	0.696	0.753	0.772	0.891	0.940	1	0.960	0.819	0.813
70°	0.728	0.746	0.749	0.852	0.886	0.960	1	0.861	0.850
80°	0.705	0.738	0.804	0.834	0.786	0.819	0.861	1	0.995
90°	0.697	0.741	0.805	0.828	0.775	0.813	0.850	0.995	1

**Table 2:** Correlation matrix of H2 Hand.

Angle	10°	20°	30°	40°	50°	60°	70°	80°	90°
10°	1	0.988	0.967	0.932	0.89	0.865	0.881	0.906	0.899
20°	0.988	1	0.984	0.949	0.904	0.872	0.89	0.914	0.905
30°	0.967	0.984	1	0.975	0.935	0.898	0.904	0.912	0.903
40°	0.932	0.949	0.975	1	0.976	0.927	0.903	0.882	0.872
50°	0.89	0.904	0.935	0.976	1	0.962	0.911	0.846	0.841
60°	0.865	0.872	0.898	0.927	0.962	1	0.961	0.827	0.831
70°	0.881	0.89	0.904	0.903	0.911	0.961	1	0.911	0.91
80°	0.906	0.914	0.912	0.882	0.846	0.827	0.911	1	0.992
90°	0.899	0.905	0.903	0.872	0.841	0.831	0.91	0.992	1

**Table 3:** PCA results for the H1 hand.

Hand H1	$\lambda_{1}$	$\lambda_{2}$	$\lambda_{3}$	$\lambda_{4}$	$\lambda_{5}$	$\lambda_{6}$	$\lambda_{7}$	$\lambda_{8}$	$\lambda_{9}$
**Eigenvalue**	7.691	0.685	0.394	0.159	0.029	0.018	0.013	0.009	0.002
**Variability (%)**	85.458	7.611	4.375	1.766	0.324	0.202	0.139	0.098	0.026
**Cumulated (%)**	85.458	93.069	97.444	99.211	99.535	99.737	99.876	99.974	100
**Eigenvector**	$F_{1}$	$F_{2}$	$F_{3}$	$F_{4}$	$F_{5}$	$F_{6}$	$F_{7}$	$F_{8}$	$F_{9}$
10°	0.318	0.477	0.123	0.58	−0.434	0.339	0.01	0.024	−0.126
20°	0.333	0.438	0.053	0.055	0.511	−0.244	−0.292	−0.456	0.277
30°	0.341	0.333	0.148	−0.315	0.293	−0.026	0.135	0.729	−0.122
40°	0.352	0.11	−0.142	−0.381	−0.262	−0.112	0.69	−0.377	−0.002
50°	0.341	−0.018	−0.434	−0.348	−0.426	−0.11	−0.606	0.05	−0.1
60°	0.332	−0.304	−0.445	0.075	0.37	0.663	0.071	−0.013	0.108
70°	0.331	−0.346	−0.254	0.529	0.071	−0.594	0.161	0.191	−0.078
80°	0.327	−0.352	0.482	−0.05	−0.241	0.037	−0.062	0.111	0.678
90°	0.325	−0.348	0.507	−0.08	0.118	0.074	−0.135	−0.256	−0.637

**Table 4:** PCA results for the H2 hand.

Hand H2	$\lambda_{1}$	$\lambda_{2}$	$\lambda_{3}$	$\lambda_{4}$	$\lambda_{5}$	$\lambda_{6}$	$\lambda_{7}$	$\lambda_{8}$	$\lambda_{9}$
**Eigenvalue**	8.296	0.333	0.23	0.083	0.028	0.011	0.009	0.006	0.003
**Variability (%)**	92.18	3.698	2.561	0.924	0.314	0.119	0.101	0.066	0.037
**Cumulated (%)**	92.18	95.878	98.439	99.363	99.677	99.796	99.897	99.963	100
**Eigenvector**	$F_{1}$	$F_{2}$	$F_{3}$	$F_{4}$	$F_{5}$	$F_{6}$	$F_{7}$	$F_{8}$	$F_{9}$
10°	0.335	0.145	−0.407	−0.439	−0.5	−0.244	−0.325	−0.256	−0.168
20°	0.338	0.121	−0.402	−0.264	0.086	0.075	0.54	0.541	0.208
30°	0.341	0.002	−0.323	0.044	0.548	0.466	−0.045	−0.492	−0.125
40°	0.338	−0.23	−0.221	0.429	0.257	−0.39	−0.497	0.344	0.132
50°	0.332	−0.43	−0.001	0.451	−0.382	−0.036	0.453	−0.141	−0.359
60°	0.327	−0.483	0.332	−0.25	−0.206	0.369	−0.181	−0.011	0.525
70°	0.332	−0.102	0.508	−0.426	0.386	−0.36	0.085	0.039	−0.39
80°	0.329	0.49	0.245	0.246	−0.014	−0.319	0.217	−0.373	0.493
90°	0.327	0.493	0.302	0.21	−0.198	0.443	−0.249	0.349	−0.309

**Table 5:** Contribution of each mode to the reconstruction of hand forces of H1.

Subject	F1	F2	F3	F4
1	0.045	−1.146	0.11	0.4
2	0.188	0.683	0.378	−0.007
3	3.344	−0.455	−0.31	−0.409
4	0.089	−0.394	0.394	0.229
5	2.716	1.034	0.254	0.979
6	4.026	0.464	−0.049	−0.155
7	−0.937	−0.198	0.23	0.073
8	−0.552	0.407	0.15	−0.214
9	−2.339	0.811	−0.001	0.111
10	1.926	−0.952	−0.71	0.37
11	5.395	−1.466	0.966	−0.66
12	−0.532	1.105	−0.156	0.369
13	2.221	1.405	−0.766	−1.007
14	2.639	−1.785	−1.987	0.543
15	3.682	0.466	1.165	0.432
16	3.526	0.628	−0.583	0.105
17	0.34	0.027	−0.712	−0.08
18	3.658	−0.804	1.471	−0.175
19	−3.343	−0.529	0.035	−0.612
20	−2.797	−0.316	−0.219	−0.161
21	−3.272	−0.183	0.272	0.07
22	−4.217	−0.673	−0.253	−0.477
23	−3.064	−0.192	0.426	0.2
24	1.234	1.677	−0.486	−0.444
25	−4.715	−0.036	0.519	0.25
26	−1.564	−0.303	0.028	−0.075
27	−0.532	1.105	−0.156	0.369
28	−3.366	−0.582	−0.017	−0.11
29	−0.693	−0.199	−0.014	0.042
30	−3.102	0.400	0.017	0.044

**Table 6:** Contribution of each mode to the reconstruction of hand forces of H2.

Subject	F1	F2	F3	F4
1	2.136	−0.559	−0.402	0.693
2	−0.011	0.105	−0.571	−0.108
3	2.582	0.009	0.091	−0.292
4	0.451	−0.114	0.033	−0.04
5	3.954	0.819	0.111	−0.214
6	3.325	−0.937	0.017	0.672
7	−0.227	−0.048	0.521	−0.025
8	−1.206	−0.701	−0.834	0.031
9	−2.118	0.232	−0.355	−0.266
10	5.828	1.059	−0.02	0.181
11	1.228	0.354	−0.169	0.061
12	−0.406	−0.034	−0.419	−0.211
13	0.959	−0.487	0.087	0.193
14	1.855	−1.058	1.546	−0.28
15	5.415	−0.599	0.142	−0.714
16	1.406	0.583	−0.724	−0.303
17	−0.066	−0.671	−0.37	−0.129
18	5.691	1.018	0.137	0.272
19	−3.46	0.434	0.482	0.45
20	−2.584	−0.49	−0.274	0.045
21	−2.72	0.65	0.671	0.08
22	−3.694	0.203	0.533	−0.129
23	−2.71	0.845	0.673	0.15
24	0.079	−0.951	0.126	−0.008
25	−4.692	0.47	−0.334	−0.197
26	−0.288	0.103	−0.532	0.145
27	−1.561	−0.094	−0.264	−0.124
28	−3.275	−0.019	−0.056	−0.264
29	−2.095	−0.111	−0.031	0.216
30	−3.795	−0.011	0.183	0.115

## Data Availability

The data presented in this study are available on request from the corresponding author. The data are not publicly available due to privacy.

## Abbreviations

The following abbreviations are used in this manuscript:

PCA	principal component analysis
PI	proximal interphalangeal joint
H1	hand number 1
H2	hand number 2
P1	phalange number 1
P2	phalange number 2
P3	phalange number 3
